# Identifying microbial signatures for patients with postmenopausal osteoporosis using gut microbiota analyses and feature selection approaches

**DOI:** 10.3389/fmicb.2023.1113174

**Published:** 2023-04-03

**Authors:** Dageng Huang, Jihan Wang, Yuhong Zeng, Qingmei Li, Yangyang Wang

**Affiliations:** ^1^Department of Spine Surgery, Honghui Hospital, Xi’an Jiaotong University, Xi’an, China; ^2^Institute of Medical Research, Northwestern Polytechnical University, Xi’an, China; ^3^Department of Osteoporosis, Honghui Hospital, Xi’an Jiaotong University, Xi’an, China; ^4^School of Electronics and Information, Northwestern Polytechnical University, Xi’an, China

**Keywords:** postmenopausal osteoporosis, gut microbiota, bone mineral density, feature selection, microbial biomarker

## Abstract

Osteoporosis (OP) is a metabolic bone disorder characterized by low bone mass and deterioration of micro-architectural bone tissue. The most common type of OP is postmenopausal osteoporosis (PMOP), with fragility fractures becoming a global burden for women. Recently, the gut microbiota has been connected to bone metabolism. The aim of this study was to characterize the gut microbiota signatures in PMOP patients and controls. Fecal samples from 21 PMOP patients and 37 controls were collected and analyzed using amplicon sequencing of the V3-V4 regions of the 16S rRNA gene. The bone mineral density (BMD) measurement and laboratory biochemical test were performed on all participants. Two feature selection algorithms, maximal information coefficient (MIC) and XGBoost, were employed to identify the PMOP-related microbial features. Results showed that the composition of gut microbiota changed in PMOP patients, and microbial abundances were more correlated with total hip BMD/*T*-score than lumbar spine BMD/*T*-score. Using the MIC and XGBoost methods, we identified a set of PMOP-related microbes; a logistic regression model revealed that two microbial markers (Fusobacteria and *Lactobacillaceae*) had significant abilities in disease classification between the PMOP and control groups. Taken together, the findings of this study provide new insights into the etiology of OP/PMOP, as well as modulating gut microbiota as a therapeutic target in the diseases. We also highlight the application of feature selection approaches in biological data mining and data analysis, which may improve the research in medical and life sciences.

## Introduction

Osteoporosis (OP) is the most common metabolic bone disease and is characterized by low bone mass and microarchitectural deterioration of bone tissue. Postmenopausal osteoporosis (PMOP), which results from estrogen deficiency, is the most common type of osteoporosis (Eastell et al., 2016). The human gut microbiota has been regarded as a key mediator of osteoporosis and osteogenesis (Seely et al., 2021). By regulating host metabolism, immune function and hormone secretion, the gut microbiota can affect bone metabolism (Li et al., 2019). For example, the gut microbiota can regulate bone mass and improve osteoporosis by inhibiting osteoclast proliferation and differentiation, inducing apoptosis, reducing bone resorption, or promoting osteoblast proliferation and maturation (Ding et al., 2020). By influencing multiple factors such as short-chain fatty acids (SCFAs), estrogen, immune factors and vitamin D, gut microbiome play important roles on calcium balance (Wang J et al., 2022). Supplementation of antibiotic-treated mice with microbial metabolism products SCFAs could induce insulin-like growth factor 1 (IGF-1) levels, and promote bone formation and growth (Yan et al., 2016). Thus, researchers have proposed that the gut microbiota is an overlooked factor that plays significant roles in osteoporosis (Hao et al., 2019). This evidence invokes the perspective of the microbiota-gut-bone axis and supports the contention that the gut microbiota may be a novel therapeutic target in the treatment of osteoporosis and fracture prevention (Li et al., 2021; He and Chen, 2022).

Recently, a growing number of studies in OP and PMOP research have linked bone loss to changes in gut microbiota. Studies have indicated that primary osteoporosis is related to changes in the gut microbiome, particularly the enriched genera *Dialister* and *Faecalibacterium* (Xu et al., 2020). Cinnamic acid suppresses bone loss *via* induction of osteoblast differentiation with the alteration of gut microbiota (Hong et al., 2022). Studies have also found that external interventions such as dietary changes can alter the gut microbiota composition and improve bone health (Yan et al., 2022). Overall, multiple factors, including hereditary, dietary and physical factors could alter the gut microbiota composition and further regulate bone metabolism (Yan et al., 2022).

Our previous studies have preliminarily investigated changes in the diversity and composition of gut microbiota in elderly Chinese osteoporosis patients (Wang et al., 2017; Wang Y et al., 2022). We found that gut microbiota changes were more highly correlated with bone mineral density (BMD) alterations in elderly females than in elderly males (Wang Y et al., 2022), which implies that the gut microbiota is a potential target for the management of PMOP patients. Here, we conducted a further analysis of the gut microbiota in postmenopausal women. It is worth mentioning that feature selection-based machine learning approaches (detailed information regarding feature selection approaches are described in the Methods) were employed to identify PMOP-related microbial features, which may provide additional clues for biological data analysis.

## Materials and methods

### Study population

A total of 58 postmenopausal female participants, including 37 healthy controls and 21 PMOP patients were recruited for this study. Specifically, female participants with a *T*-score > −2.5 for the BMD measurement were considered as healthy controls, whereas female participants with a *T*-score < −2.5 for the BMD measurement were diagnosed with PMOP. We excluded participants or patients with other malignancies, including chronic heart disease, liver disease, kidney disease, diabetes, and diseases related to secondary OP (hyperthyroidism, steroid abuse, Cushing’s syndrome, hyperparathyroidism, etc.,). In this study, none of the participants had a history of fractures. The participant characteristics are shown in Table 1. Our study was approved by the Institutional Review Board of Honghui Hospital, Xi’an Jiaotong University and was a part of the project “Diversity Analysis for Intestinal Flora in Patients with Primary Osteoporosis” registered at ^1^ as #ChiCTR-1,800,019,048#. Written informed consent was obtained from each participant.

**Table 1:** Characteristics of the 58 participants and the statistical results between PMOP and control groups.

Characteristics	Total (*n* = 58)	PMOP (*n* = 21)	Control (*n* = 37)	*p* value
**Body measurements**				
Age (*y*)	57.47 ± 5.32	59.14 ± 4.40	56.91 ± 5.62	0.0567
Menopause age (*y*)	47.59 ± 2.36	47.71 ± 2.53	47.51 ± 2.27	0.9607
Waist circumference (cm)	84.61 ± 8.88	83.85 ± 5.47	85.04 ± 10.37	0.6978
BMI (kg/m^2^)	23.43 ± 3.07	22.59 ± 2.24	23.91 ± 3.39	0.1242
**Biochemical tests**				
Ca (mmol/L)	2.28 ± 0.28	2.21 ± 0.35	2.32 ± 0.23	0.5172
P (mmol/L)	1.18 ± 0.18	1.16 ± 0.23	1.20 ± 0.16	0.7522
Vitamin D (ng/mL)	18.43 ± 6.95	19.41 ± 9.63	17.87 ± 4.90	0.7297
ALP (U/L)	98.31 ± 30.20	95.90 ± 35.51	99.68 ± 27.17	0.8587
b-CTX (ng/mL)	0.43 ± 0.14	0.45 ± 0.14	0.43 ± 0.14	0.5878
Total P1NP (ng/mL)	74.05 ± 28.31	72.77 ± 33.61	74.78 ± 25.30	0.7340
**BMD measurements**				
Lumbar spine BMD (g/cm^2^)	0.79 ± 0.13	0.66 ± 0.08	0.86 ± 0.10	**< 0.0001**
Lumbar spine T-score	−1.79 ± 1.12	−2.85 ± 0.70	−1.18 ± 0.83	**< 0.0001**
Total hip BMD (g/cm^2^)	0.65 ± 0.12	0.55 ± 0.08	0.71 ± 0.09	**< 0.0001**
Total hip T-score	−1.63 ± 1.05	−2.53 ± 0.77	−1.12 ± 0.81	**< 0.0001**

PMOP: Postmenopausal Osteoporosis; BMI: Body Mass Index; ALP: Alkaline Phosphatase; b-CTX: C-Terminal telopeptide of type I collagen; P1NP: Procollagen type 1 N-peptide; BMD: Bone Mineral Density. The *p* value was obtained from Wilcoxon test in *R*. The bold *p* values showed significant statistical results.

### Biochemical tests

Fasting venous blood samples were collected from the 58 participants for laboratory biochemical analysis, including serum calcium (Ca), phosphorus (P), 25-hydroxyvitamin D (vitamin D), alkaline phosphatase (ALP), C-terminal telopeptide of type I collagen (b-CTX) and procollagen type 1 N-peptide (P1NP). A 5 ml vacuum blood collection tube was used to collect fasting venous blood, which was left at room temperature for 45 (±10) min, and the serum was collected for detection after centrifugation (not less than 1.5 mL serum). Specifically, the serum contents of ALP were detected by using a Roche Cobas c501 automatic biochemical analyzer and the corresponding reagents (Roche, United States); serum contents of Ca, P, Vitamin D, b-CTX and P1NP were detected by using a Roche Cobas e601 electrochemiluminescence instrument and the corresponding reagents (Roche, United States).

### BMD measurement

All 58 participants underwent BMD measurement with a HOLOGIC Discovery Dual Energy X-ray Absorptiometry (DXA) scanner (HOLOGIC, United States). Subjects meeting any one of the following criteria were not allowed to undergo BMD measurement: (1) patients who had taken orally administered drugs within the previous 2–6 d that could affect image development, (2) patients who were physically weak and unable to lie on their backs or lie flat for 5 min, (3) patients with metallic implants or severe deformities of the spine, or (4) patients who had radioisotope examinations within the previous 3 days. For all participants, both lumbar spine and total hip BMD measurements were performed. We collected information pertaining to the participant BMD values (g/cm^2^) and *T*-scores of the lumbar spine and total hip. The T-score reference ranges were calculated with data from a healthy Asian population provided by the bone densitometry equipment manufacturer. Based on the diagnostic criteria, postmenopausal females with *T*-scores ≤ −2.5 at any site were diagnosed as having PMOP, −2.5 < *T*-scores < −1.0 were diagnosed as having osteopenia (ON), and *T*-scores ≥ −1.0 indicated normal bone mass. In this study, we divided the subjects into two major groups according to the diagnostic criteria: the PMOP group (*T*-scores ≤ −2.5 at any site) and the control group (*T*-scores > −2.5 at any site).

### Fecal sample collection and bacterial DNA extraction

We collected 58 fecal samples from the participants for gut microbiota analyses. Before the collection of fecal samples, the subjects were required to avoid the following medicines for 1 month: antibiotics, probiotic, steroids, nonsteroidal anti-inflammatory drugs, microecological adjustment preparations, immunosuppressants, and any Chinese herbal medicine preparations. Participants were required to collect a 3–5 mL fresh stool sample between 6:00 and 8:00 in the morning on the day of fecal sample collection using a disposable sterile stool collection tube. The collected sample collection tube was placed in an ice box, and the researcher transported the sample to the laboratory within 2 h after collection and stored it at −80°C until further use. The QIAamp Fast DNA Stool Mini Kit (Qiagen, Germany) was used to extract the microbial genome from fecal samples following the manufacturer’s instructions. DNA concentration and purity were monitored on 1% agarose gels. Based on the concentration, the DNA was diluted to 1 ng/μL using sterile water.

### 16S rRNA polymerase chain reaction (PCR)

The TransGen AP221-02 Kit (TransGen, China) was used to amplify the V3-V4 region of the microbial 16S RNA gene. The polymerase chain reaction (PCR) primers of the 16S rRNA V3-V4 region were as follows: 338F 5’-ACTCCTACGGGAGGCAGCAG-3′ and 806R 5’GGACTACHVGGGTWTCTAAT-3′(Mori et al., 2014). The same volume of 1× loading buffer (containing SYBR green) was mixed with the PCR products, and electrophoresis was performed on a 2% agarose gel for detection. The PCR products were mixed in equidensity ratios. Next, the PCR products were purified with a GeneJET Gel Extraction Kit (Thermo Scientific, United States).

### Library preparation and Illumina sequencing

The sequencing libraries were generated using the NEBNext Ultra DNA Library Prep Kit for Illumina (New England Biolabs, United States) following the manufacturer’s recommendations. The library quality was assessed on a Qubit@ 2.0 Fluorometer (Thermo Scientific, United States). Finally, the library was sequenced on the Illumina NovaSeq platform (Illumina, United States). The 16S rRNA amplicon sequencing project for 58 participants is available on the NCBI-BioProject site under accession PRJNA565497, and the raw data have been deposited in the NCBI-SRA database (accessions SRX6849522-SRX6849524, SRX6849535, SRX6849539-SRX6849588, SRX6849591, SRX6849602, SRX6849613, and SRX6849624).

### Data analysis for 16S rRNA amplicon sequencing

Sequence analysis was performed with UPARSE software (UPARSE v7.0.1001, http://drive5.com/uparse/) (Edgar, 2013). Sequences with ≥97% similarity were assigned to the same operational taxonomic units (OTUs). Representative sequences for each OTU were screened for further annotation. For each representative sequence, the Silva Database (Version 138, https://www.arb-silva.de/) (Quast et al., 2013) was used to annotate taxonomic information based on the Mothur Bayesian classifier algorithm. To study the phylogenetic relationships of different OTUs and the difference in the dominant species among samples (groups), multiple sequence alignment was conducted using MUSCLE software (Version 3.8.31, http://www.drive5.com/muscle/). OTU abundance information was normalized using a standard sequence number corresponding to the sample with the fewest sequences. Alpha diversity was used to analyze the complexity of species diversity for a sample through several indices, including observed species, Shannon, Simpson, Chao1 and Ace. All of these indices were calculated with QIIME (Version 1.7.0). Specifically, the Shannon and Simpson indices were used to identify community diversity; the Chao1 and Ace indices were selected to identify community richness. For beta diversity analysis, we calculated both weighted and unweighted UniFrac distances using QIIME for principal coordinate analysis (PCoA).

### Using XGBoost and SHAP methods to identify microbial biomarkers for PMOP

To obtain gut microbes (as features) that have the most significant effect on PMOP, the methods of maximal information coefficient (MIC) (Reshef et al., 2011; Kinney and Atwal, 2014) and XGBoost (Chen et al., 2015; Chen and Guestrin, 2016) explained with Shapley Additive exPlanations (SHAP) (Lundberg and Lee, 2017; Lundberg et al., 2020; Parsa et al., 2020) were employed for feature selection, and the intersection of the two sets of results was selected as the final microbial feature.

### Maximal information coefficient

Reshef, etc., proposed a novel correlation measurement algorithm designated MIC based on mutual information (MI). Compared with MI, MIC is more equitable for high-dimensional datasets and has a wider range of applications in feature selection. For a dataset D , the number of samples is n , and x,y are positive integers greater than 1. The MIC can be computed by:


(1) $$MIC\left(D\right)=\underset{xy<B}{\max}\frac{I^{*}\left(D,x,y\right)}{\log_{2}\min\left(x,y\right)}$$

where $B\left(n\right)=n^{0.6}$ , ${I^{*}\left(D,x,y\right)=\max_{G}I(D|}_{G})$ and $I\left({D|}_{G}\right)$ is the MI of D in the grid of G . The correlations between features and classifications can be measured by MIC, and those features with larger MIC values were considered potential signatures for the identification of PMOP. The MIC code is available on https://github.com/minepy/minepy. In our study, we performed the MATLAB version to obtain the associations between any two variables, and the parameter of alpha for function “mine” was set to 0.6.

### XGBoost explained with SHAP

XGBoost is an efficient implementation of gradient-boosted decision trees that uses a sequence of decision trees to improve the model and build a stronger learner (Parsa et al., 2020). The objective function L(ϕ) of XGBoost is as follows:


(2) $$\begin{array}{l} L\left(\phi \right)=\overset{n}{\underset{i=1}{\sum }}l\left({\overset{}{\hat{y}}}_{i},y_{i}\right)+\underset{k}{\sum }\Omega \left(f_{k}\right)\\ where\Omega \left(f\right)=\gamma T+\frac{1}{2}\lambda ||\omega |{|}^{2} \end{array}$$

L(ϕ) is composed of two parts: a convex loss function l and a regularization term Ω . The loss function measures the difference between the prediction ${\overset{}{\hat{y}}}_{i}$ and the target $y_{i}$ , and the regular function penalizes the complexity of the model (Chen and Guestrin, 2016). The parameters of γ and λ are set manually; ω is the vector formed by the values of all leaf nodes of the decision tree, and T is the number of leaf nodes.

Generally, models using XGBoost are considered black boxes due to their complexity, and the interpretability of the XBGoost model is critical to the prediction accuracy for many applications such as bioinformatics and medicine. In 2017, Lundberg et al. proposed Shapley Additive exPlanations (SHAP) (Lundberg and Lee, 2017; Lundberg et al., 2020), a unified framework for interpreting predictions, for interpreting the output of those black boxes, and the importance of each feature can be computed using the TreeExplainer of SHAP with a high speed (Lundberg et al., 2020). In our study, we used TreeExplainer to obtain the most valuable factors with the greatest impact on PMOP. The versions of Shap and XGBoost based on Python 3.9.16 are 0.41.0 and 1.6.2, respectively. The code using shap and XGBoost to explain the output is available on.^2^

### Statistical analysis

The statistical tools R 3.6.0 and GraphPad Prism 5.01 were used for statistical analysis and figure generation in this study. Data on body measurements, biochemical tests, BMD measurements, and alpha diversity indices are presented as the mean ± standard deviation (SD). Differential analyses between the PMOP and control groups of body measurements, biochemical tests, BMD measurements, and alpha diversity indices were performed with the Wilcoxon test in R, with *p* < 0.05 indicating a significant difference. For gut microbiota composition, we collected the top 10 dominant microbial taxa at the phylum, class, order, family, genus, and species levels, with *p* < 0.05 in the Wilcoxon test indicating a significant difference between the PMOP and control groups and 0.05 < *p* < 0.1 indicating a tendency toward differences. Correlations between gut microbial composition and BMD value/*T*-score as well as between the abundance of functional group and BMD value/*T*-score were performed using the Spearman correlation test in R, with *p* < 0.05 indicating significant correlations and 0.05 < *p* < 0.1 indicating a tendency for correlations. MedCalc 20.104 and GraphPad Prism 5.01 statistical software were used for logistic regression and receiver operating characteristic (ROC) analysis based on the gut microbiota.

## Results

### Characteristics of the study population

The baseline demographic features, laboratory biochemical tests and bone BMD measurements of the participants are summarized in Table 1. Based on the diagnostic and exclusion criteria, a total of 58 postmenopausal female participants were included in this study. The average age and menopausal age of 58 participants were 57.47 ± 5.32 and 47.59 ± 2.36, respectively. Generally, there were no significant differences in baseline demographic features or biochemical tests between the PMOP and control groups (*p* > 0.05 in the Wilcoxon test). However, BMD values and T-scores for both the lumbar spine and total hip of PMOP subjects were significantly lower than those of the control subjects (*p* < 0.0001). Detailed information regarding demographic features, laboratory biochemical tests and BMD measurements are described in the Methods.

### Gut microbial diversity and composition analysis between the PMOP and control groups

To identify the gut microbiota features of the participants and microbial composition changes in patients with PMOP, we sequenced and analyzed the 16S rRNA V3-V4 region of fecal samples from the participants using the Illumina platform. As described in the Methods, we deposited the raw data of the sequencing files into the NCBI-SRA database. The species accumulation curve of 58 samples tended to plateau with increasing sample size, which indicates that the amount of measured data was reasonable and reached the sequencing depth, as shown in Supplementary Figure S1. Generally, no significant differences in the richness and diversity of the gut microbiota were found between PMOP and control subjects, although the control group exhibited slightly higher values of observed species and higher indices of Chao1 and Ace than those of the PMOP group (*p* > 0.1, Supplementary Table S1). To assess the overall structural changes in the gut microbiota between the PMOP and control groups, PCoA score plots were constructed based on both weighted and unweighted UniFrac distances. As shown in Supplementary Figure S2, the permutational multivariate analysis of variance (PerMANOVA) test based on weighted (*p* = 0.329) and unweighted UniFrac distance measures (*p* = 0.025) showed that the microbial structure differed between the PMOP and control subjects.

**Figure 1:**
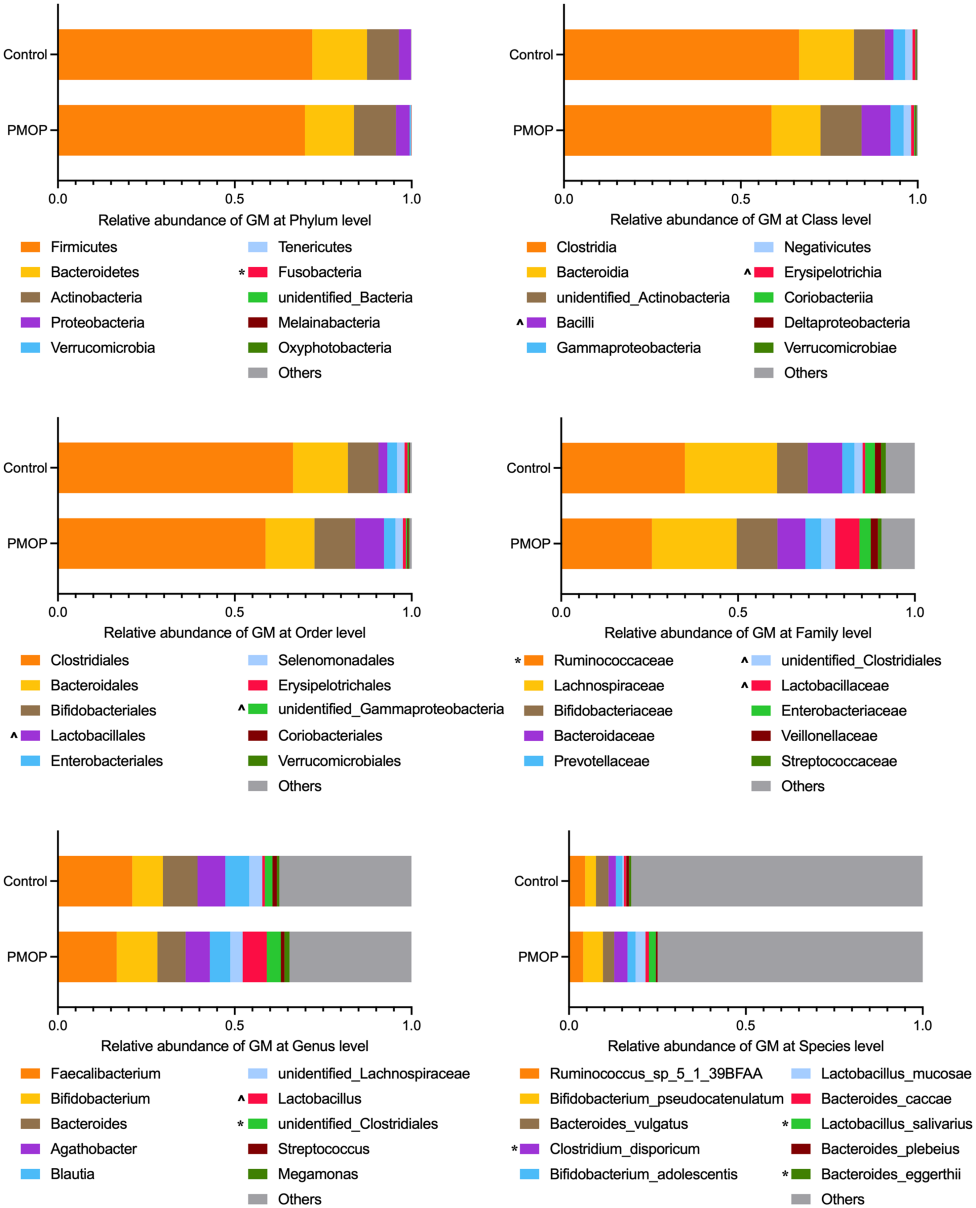
Relative abundance of gut microbiota at different taxonomy levels. The statistical analysis was performed using Wilcoxon test in *R*, “*” represents *p* < 0.05, “^” represents 0.05 < *p* < 0.1. Detailed values about the boxplot were summarized in Supplementary Table S2.

We then identified the gut microbial compositions in all the samples. The microbial community analysis indicated that Firmicutes, Bacteroidetes, Actinobacteria and Proteobacteria accounted for the majority of the phyla with relative abundances of 71.16, 14.91, 10.02, and 3.59%, respectively, in the total study population. There were no significant differences in the abundances of the four major phyla between the PMOP and control groups (*p* > 0.1), although the PMOP subjects exhibited relatively higher Fusobacteria abundance than that of the control subjects (*p* = 0.0395). Figure 1 and Supplementary Table S2 illustrate and summarize the relative microbial compositions in PMOP and control subjects at the phylum, class, order, family, genus and species levels. Notably, the microbial compositions of Bacilli and Erysipelotrichia were higher in the PMOP group than those in the control group (*p* < 0.1 at the class level), and the relative abundances of Lactobacillales/*Lactobacillaceae*/*Lactobacillus*/*Lactobacillus salivarius* were also increased in PMOP subjects at the order/family/genus/species levels, respectively (*p* < 0.1). In contrast, the compositions of the bacteria Ruminococcaceae and *Bacteroides eggerthii* were lower in the PMOP group than those in the control group at the family and species levels (*p* < 0.05), respectively.

### Gut microbial compositions were more correlated with the BMD value/*T*-score of the total Hip

**Figure 2:**
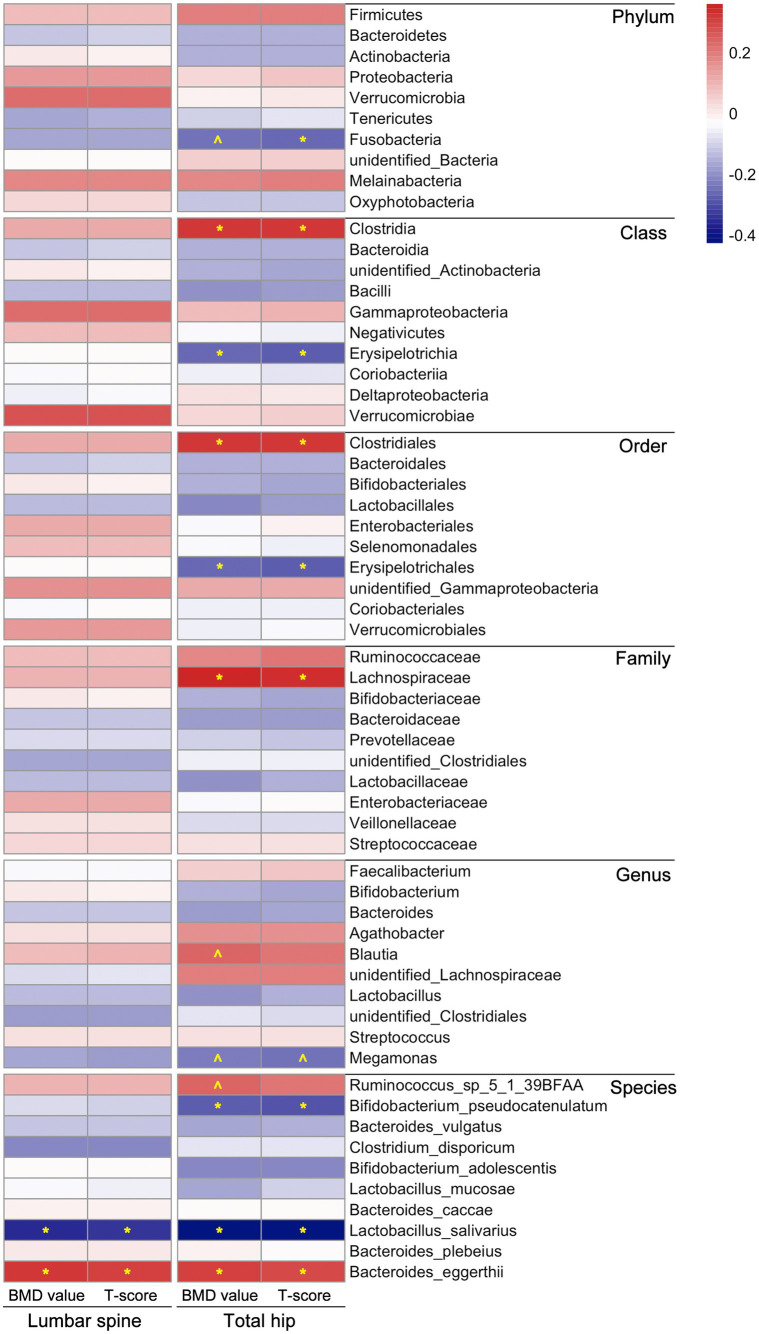
Correlations between microbial composition and BMD value/T-score at both lumbar spine and total hip of the participants. The statistical analysis was performed using Spearman correlation test in *R*, “*” represents *p* < 0.05, “^” represents 0.05 < *p* < 0.1; different colors represent the Spearman correlation coefficients. Detailed values about the correlation analysis were summarized in Supplementary Table S3.

We subsequently assessed the correlation between gut microbiota composition and BMD value or T-score to further explore BMD-related microbiota in postmenopausal females. It is worth mentioning that there were more correlations between microbial composition and BMD value/*T*-score observed in the total hip than in the lumbar spine, as demonstrated through Spearman correlation coefficients. In the lumbar spine, only the abundances of two species, *Lactobacillus salivarius* and *Bacteroides eggerthii*, were correlated with BMD value/*T*-score of the participants (*p* < 0.05). In the total hip, the relative abundances of microbial taxa, including Clostridia/Clostridiales, Lachnospiraceae, *Blautia*, *Streptococcus*, *Ruminococcus*_sp_5_1_39BFAA and *Bacteroides eggerthii*, were positively correlated with BMD value/*T*-score; whereas Fusobacteria, Erysipelotrichia/ Erysipelotrichales, Megamonas, *Bifidobacterium pseudocatenulatum* and *Lactobacillus salivarius* compositions were negatively correlated with the BMD value/T-score of the participants, detailed results of the Spearman correlation analysis were shown in Figure 2 and Supplementary Table S3.

### Feature selection methods identified microbial biomarkers for PMOP

**Figure 3:**
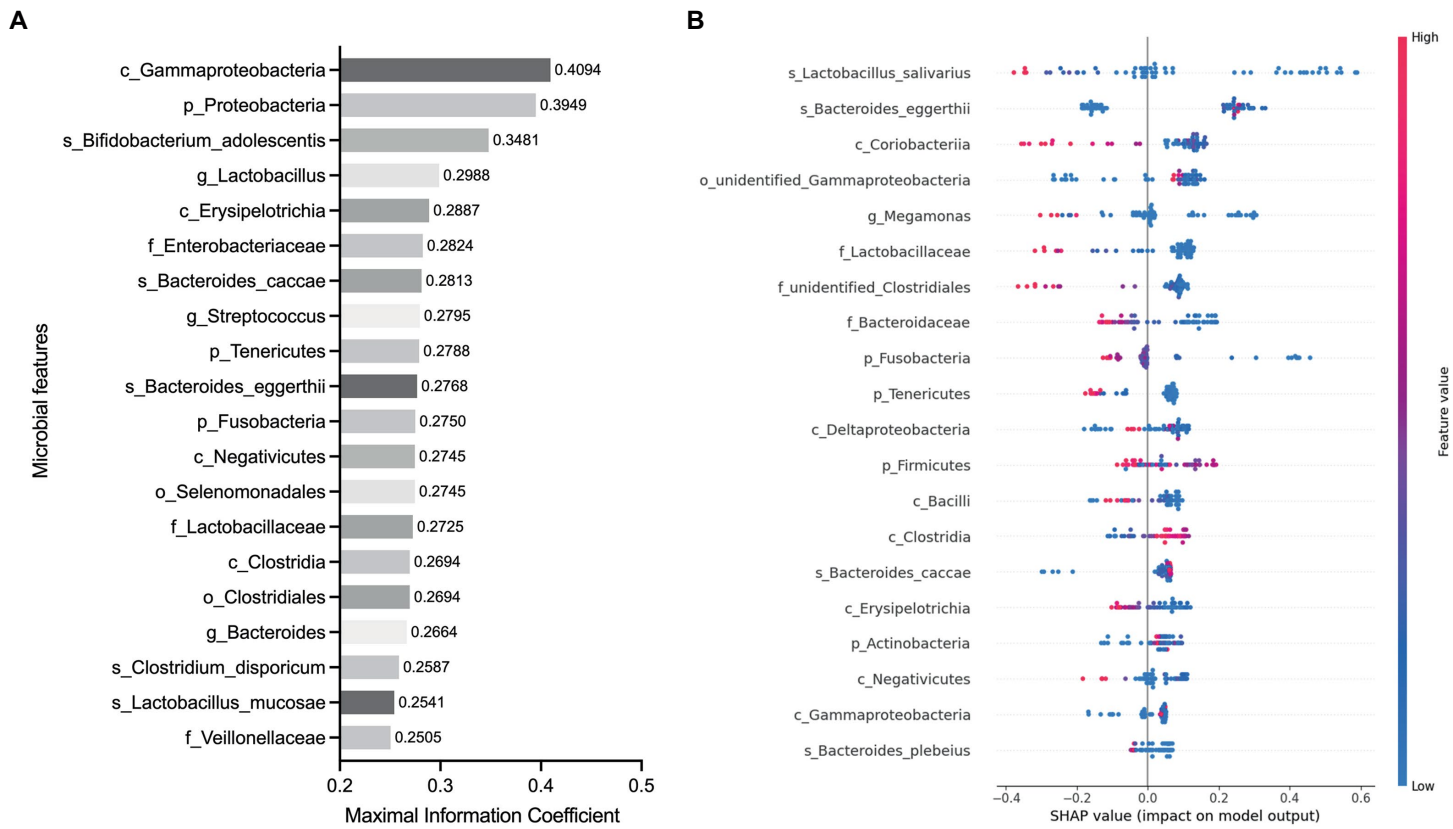
Identified the top 20 microbial features as the classification biomarkers by using feature selection methods. **(A)** Top 20 microbial features selected by MIC method. **(B)** Top 20 microbial features selected by XGBoost explained with SHAP. Each point represents a sample, the color represents the feature value (red high, blue low).

To determine whether the gut microbiota can be regarded as an identifying biomarker for distinguishing PMOP from control subjects, two major feature selection methods, the MIC and XGBoost explained with SHAP, were employed, and ROC curves were utilized to test the performance of the classification (details are provided in the Methods). In the feature selection procedure, we analyzed the importance of the 60 major microbial features (as biomarkers in classifying the PMOP and control groups) at all six taxonomic levels and selected the top 20 microbial features based on both the MIC and XGBoost approaches. The maximal information coefficient is a measure of the strength of the linear or nonlinear association between microbes and PMOP, and we identified the top 20 PMOP-related microbes with MIC, as shown in Figure 3A. SHA*p* values of the top 20 microbial features for every sample are illustrated in Figure 3B. The plot below sorts features by the sum of the SHAP value magnitudes over all samples and uses SHAP values to show the distribution of the impacts that each feature has on the model output. We identified nine microbial features that overlapped between the two feature selection approaches, including the phyla Tenericutes and Fusobacteria; classes Clostridia, Gammaproteobacteria, Negativicutes, and Erysipelotrichia; family *Lactobacillaceae*, and species *Bacteroides caccae* and *Bacteroides eggerthii*. Logistic regression results of the nine microbes indicated that two microbial markers, Fusobacteria and *Lactobacillaceae*, had significant abilities in disease classification between the PMOP and control groups; the logistic regression equation was Y = −2.920 + 4412*(p_Fusobacteria) + 34.75*(f_*Lactobacillaceae*) (Table 2). We could distinguish PMOP patients from control subjects based on the combination of p_Fusobacteria and f_*Lactobacillaceae*, as indicated by the area under the ROC curve (AUC), which had a value of 0.7709 (*p* < 0.0001, Std. Error: 0.0687, 95% CI: 0.642 to 0.871, Supplementary Figure S3). Overall, the PMOP-associated microbial features captured by the feature selection method offered further evidence of the dysbiotic gut microbiome and highlighted its potential application for the detection of PMOP.

**Table 2:** Logistic regression results of the selected microbial features in disease classification.

Variable	Coefficient	Std. Error	95% CI	Wald	*p*
Constant	−2.920	0.9312	−5.057 to −1.344	9.8337	**0.0017**
p_ Fusobacteria	4412	2084	956.5 to 9,151	4.4816	**0.0343**
f_ *Lactobacillaceae*	34.75	18.29	10.11 to 84.25	3.6107	**0.0574**

The bold *p* values showed significant statistical results.

## Discussion

In the current study, we demonstrated that PMOP patients had different gut microbiota profiles compared with those of control subjects and showed that the compositions of microbiota were more correlated with BMD values of the total hip than those of the lumbar spine in participants. By conducting feature selection, we identified PMOP-related microbes, which could be used as biomarkers for PMOP diagnosis or management.

Our results confirmed that gut microbiota composition were altered in PMOP patients compared with those in normal patients. The abundance of Fusobacteria increased in the PMOP patients (PMOP vs. control, 0.08% vs. 0.03%, *p* = 0.0395). Consistent with other studies, the relative abundance of Fusobacteria was negatively associated with the BMD value (Greenbaum et al., 2022). Fusobacteria has been shown to promote M1 macrophage production *via* AKT2 signaling (Liu et al., 2019), which induces inflammation and prompts the development of osteoporosis (Yang and Yang, 2019). In another randomized trial of postmenopausal Japanese women, the total hip BMD of participants increased after receiving the probiotic *Bacillus subtilis* (C-3102) compared with that of the placebo group (Cronin et al., 2022). Consistent decreases in Fusobacteria species were noted at 12 and 24 weeks after using C-3102, which led the researchers to speculate that the decrease in Fusobacteria might favorably influence BMD by decreasing the production of cytokines that regulate bone resorption (Cronin et al., 2022). In addition, we also observed higher levels of *Lactobacillales*/*Lactobacillaceae*/*Lactobacillus*/*Lactobacillus salivarius* in PMOP patients than those in the control group, which is contradictory to other results and requires further confirmation. In most related studies, supplementation with probiotics increased BMD values (Yu et al., 2021). For example, supplementation with *Lactobacillus reuteri* ATCC PTA 6475 reduced bone loss in older women with low BMD (Li et al., 2022). *Lactobacillus acidophilus* inhibited bone loss and increased bone heterogeneity by modulating Treg-Th17 cell function in osteoporotic mice (Dar et al., 2018). *Lactobacillus rhamnosus* attenuated bone loss and maintained bone health in ovariectomized mice (Sapra et al., 2021). The above evidence supports the beneficial effects of *Lactobacillales*/*Lactobacillaceae*/*Lactobacillus* on increasing BMD in osteoporosis patients and mouse models. It is worth mentioning that even within the same phylum, each taxon such as family, genus or species has different functions (Ozaki et al., 2021). Generally, *Lactobacillales*/*Lactobacillaceae*/*Lactobacillus* bacteria contribute to weight loss and thus benefit obesity treatment, and studies have shown that the beneficial effects are strain dependent (Crovesy et al., 2017; Alvarez-Arrano and Martin-Pelaez, 2021). *Lactobacillus* can reduce body weight and alleviate fat accumulation in mice fed a high-fat diet (Wang et al., 2020). For osteoporosis patients, increasing BMI was identified as a protective factor for bone loss (Wang Y et al., 2022; Yin et al., 2022). The results from a large cohort survey showed that the mean rate of fragility fracture was significantly increased in the underweight group compared with the obese and normal weight groups in postmenopausal women (Kim et al., 2021). In an age-adjusted analysis, men who lost weight during follow-up had a significantly greater rate of BMD loss (Sheu et al., 2009). Thus, we may initially obtain a negative correlation between *Lactobacillales*/*Lactobacillaceae*/*Lactobacillus* and body weight or BMI as well as a positive correlation between BMD and body weight or BMI, within a reasonable range. Although this does not represent a negative correlation between *Lactobacillales*/*Lactobacillaceae*/*Lactobacillus* and BMD value, as discussed earlier, most studies have shown that *Lactobacillales* or *Lactobacillus* is beneficial for increasing bone mass, and therefore we may speculate that different species of *Lactobacillales*/*Lactobacillaceae*/*Lactobacillus* have different functions, and some strains may be unfavorable for increasing bone mass. Subsequent validation with larger populations and mechanistic experiments should be performed to better explore this hypothesis.

In contrast to the elevated microbial taxonomies, the abundances of Ruminococcaceae (PMOP vs. control, 25.64% vs. 34.99%, *p* = 0.0332) and *Bacteroides eggerthii* (PMOP vs. control, nearly 00.00% vs. 0.62%, *p* = 0.0007) were lower in PMOP patients than those in control subjects. The bacteria of Ruminococcaceae include some butyric acid-producing microbes such as *Faecalibacterium* and *Butyricicoccus* (Ozaki et al., 2021). Butyrate-producing bacteria are promising probiotic candidates for gastrointestinal disorders such as inflammatory bowel disease (IBD) (Roux et al., 2015). A recent study demonstrated that *Bacteroides eggerthii* was decreased in OP patients and negatively correlated with bone resorption markers but positively correlated with bone formation markers and 25-OH-D3 (Qin et al., 2021), which is consistent with our findings in the current study.

We performed BMD measurements at both the lumbar spine and total hip for all the participants in this study. The Spearman correlation analysis revealed that gut microbial compositions were more related to BMD value/*T*-score at the total hip than those at the lumbar spine. Researchers have suggested that clinicians and densitometrists should expect that more than 40% of women screened for DXA will have T-score discordance between the spine and hip, which is an important point worthy of attention (Yoon and Kim, 2021). In clinical trials, changes in BMD have been shown to provide a reliable estimate of treatment-related antifracture effects, and evidence supports the suitability of total hip BMD as a meaningful outcome for the clinical management of patients with OP (Leslie et al., 2016; Banefelt et al., 2022). In another earlier cohort study, the total hip was the best site for overall fracture assessment, and the spine was the most useful site for the prediction of spinal fractures alone (Leslie et al., 2007). The above evidence and our findings indicate that the BMD value/*T*-score and BMD-related factors (such as BMD-related microbiota) are discordant among different test sites. This phenomenon should be regarded as a real and prevalent finding for physicians and researchers to develop better strategies for patients (Moayyeri et al., 2005; Leslie et al., 2020).

In the procedure of identifying PMOP-related microbial biomarkers, we highlight the application of feature selection approaches for biological data mining and analysis. Recently, the number of microbiome-related studies has notably increased the availability of data on human microbiome composition and function, which has prompted the application of machine learning in human microbiome studies, including but not limited to feature selection, biomarker identification, disease prediction and treatment (Goren et al., 2021; Marcos-Zambrano et al., 2021; Hinton and Mucha, 2022). Recently, human gut microbiota biomarkers were selected *via* different feature selection methods for patients with IBD (Bakir-Gungor et al., 2022). In addition to gut microbes (Cai et al., 2015; Ai et al., 2019), advanced feature selection methods also worked well for selecting the key gut microbiome genes (Chen et al., 2016). In this study, we selected several gut microbes that were most strongly related to PMOP *via* the feature selection methods MIC and XGBoost and further demonstrated that the combination of Fusobacteria and *Lactobacillaceae* performed well in distinguishing between the PMOP and control groups. We highlight the power and utility of feature selection methods in data mining and processing during biological dataset analysis for identifying diagnostic or predictive markers.

Our study has several limitations. Firstly, the GM analyses associated with this study is limited to its small sample size, which directly influences the research findings. Besides, the research was not profound enough, particularly in relying solely on fecal sample 16S rRNA sequencing to study the intestinal flora. Moreover, multiple factors such as the dietary habit, physical activity, smoking habit influent the GM structure and composition, it’s necessary to consider the confounders of individuals in larger cohort in the future study, to make the research more rigorous.

## Conclusion

OP is a chronic, long-term pathologic process that is associated with heredity and environmental factors. PMOP is the most common type of OP and has become a major public health burden for women around the world. Evidence has demonstrated the linkages between OP/PMOP and gut microbiota. Our results showed that gut microbial compositions were altered in the PMOP group compared with the control group. However, the richness and diversity of the gut microbiota between the two groups were not that different. We performed BMD measurements at both the lumbar spine and total hip for all the participants. The Spearman correlation analysis revealed that gut microbial compositions were more strongly related to BMD value/T-score at the total hip than the lumbar spine. Furthermore, we employed two major feature selection methods, MIC and XGBoost, to identify PMOP-related microbial biomarkers, and the combination of specific microbes exhibited potential diagnostic value for distinguishing between PMOP and normal samples. These findings may provide new insights for revealing potential etiologies for OP/PMOP, understanding the role of gut microbiota and modulating gut microbiota as a therapeutic target in the diseases. In addition, we highlight the application of feature selection approaches in biological data mining and data analysis, which may help improve the research techniques for the medical and life sciences.

## Data availability statement

The datasets presented in this study can be found in online repositories. The names of the repository/repositories and accession number(s) can be found in the article/Supplementary material.

## Ethics statement

The studies involving human participants were reviewed and approved by the Institutional Review Board of Honghui Hospital, Xi’an Jiaotong University (no. 201801019); and was a part of the project “Diversity Analysis for Intestinal Flora in Patients with Primary Osteoporosis” registered at www.chictr.org.cn as #ChiCTR-1,800,019,048#. The patients/participants provided their written informed consent to participate in this study.

## Author contributions

DH and JW: project administration and funding acquisition. YZ: cases inclusion and exclusion, blood sample collection, and preparation. YW: data processing and analysis, manuscript writing, and supervision. QL: manuscript revision. All authors contributed to the article and approved the submitted version.

## Funding

This work was by supported by National Natural Science Foundation of China (no. 81702067); Shaanxi Provincial Key Research and Development Program (np. 2020GXLH-Y-027; no. 2021SF030); Fundamental Research Funds for the Central Universities (G2020KY0516).

## Conflict of interest

The authors declare that the research was conducted in the absence of any commercial or financial relationships that could be construed as a potential conflict of interest.

## Publisher’s note

All claims expressed in this article are solely those of the authors and do not necessarily represent those of their affiliated organizations, or those of the publisher, the editors and the reviewers. Any product that may be evaluated in this article, or claim that may be made by its manufacturer, is not guaranteed or endorsed by the publisher.

## Footnotes

^1^ www.chictr.org.cn

^2^ https://github.com/slundberg/shap
