# Supplementary figures and images for: Identifying microbial signatures for patients with postmenopausal osteoporosis using gut microbiota analyses and feature selection approaches
Source: Front Microbiol. 2023 Apr 3;14:1113174. doi: 10.3389/fmicb.2023.1113174 (PMC10106639; doi:10.3389/fmicb.2023.1113174)

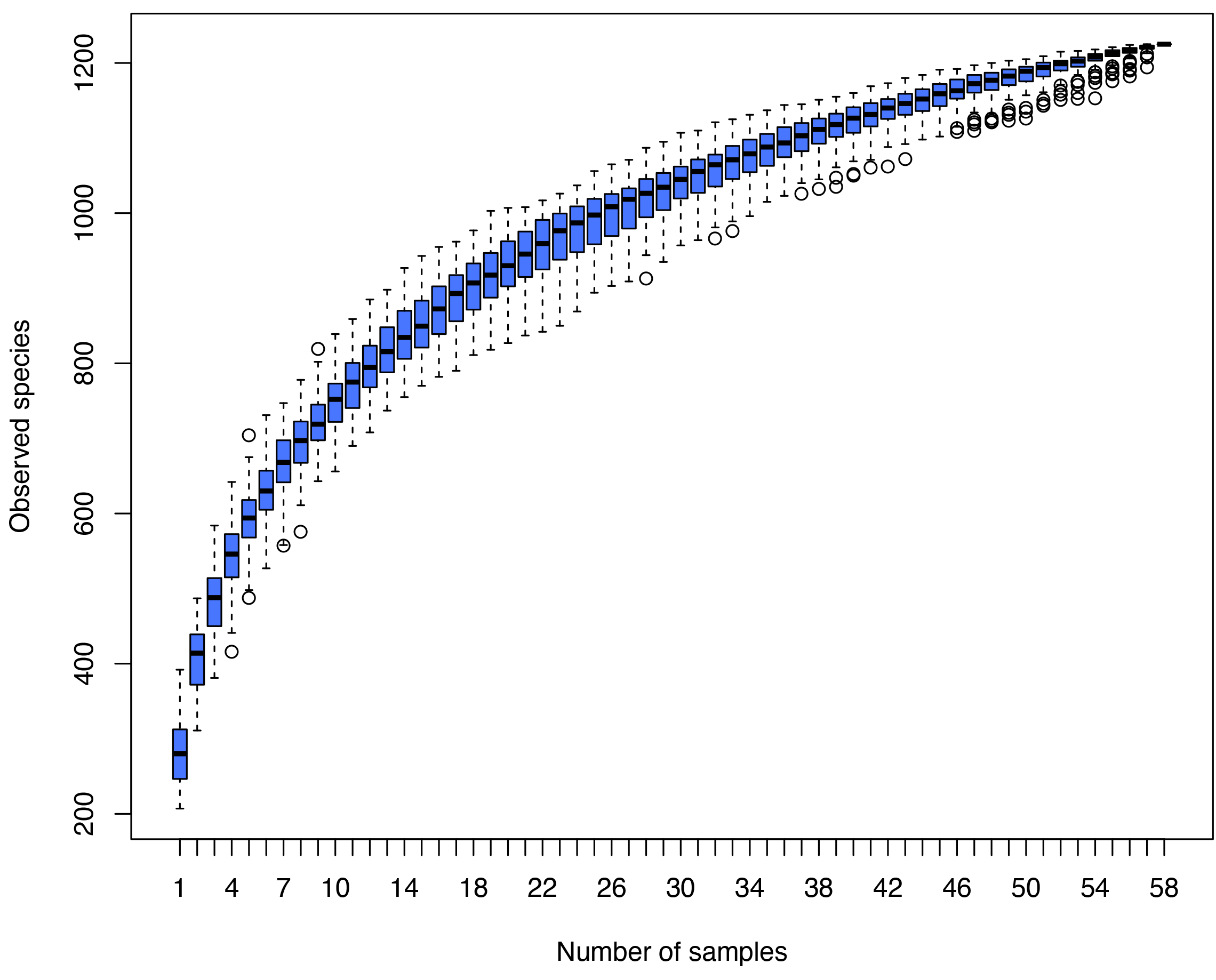

Supplement: Supplementary file 1 [file Image_1.TIF]

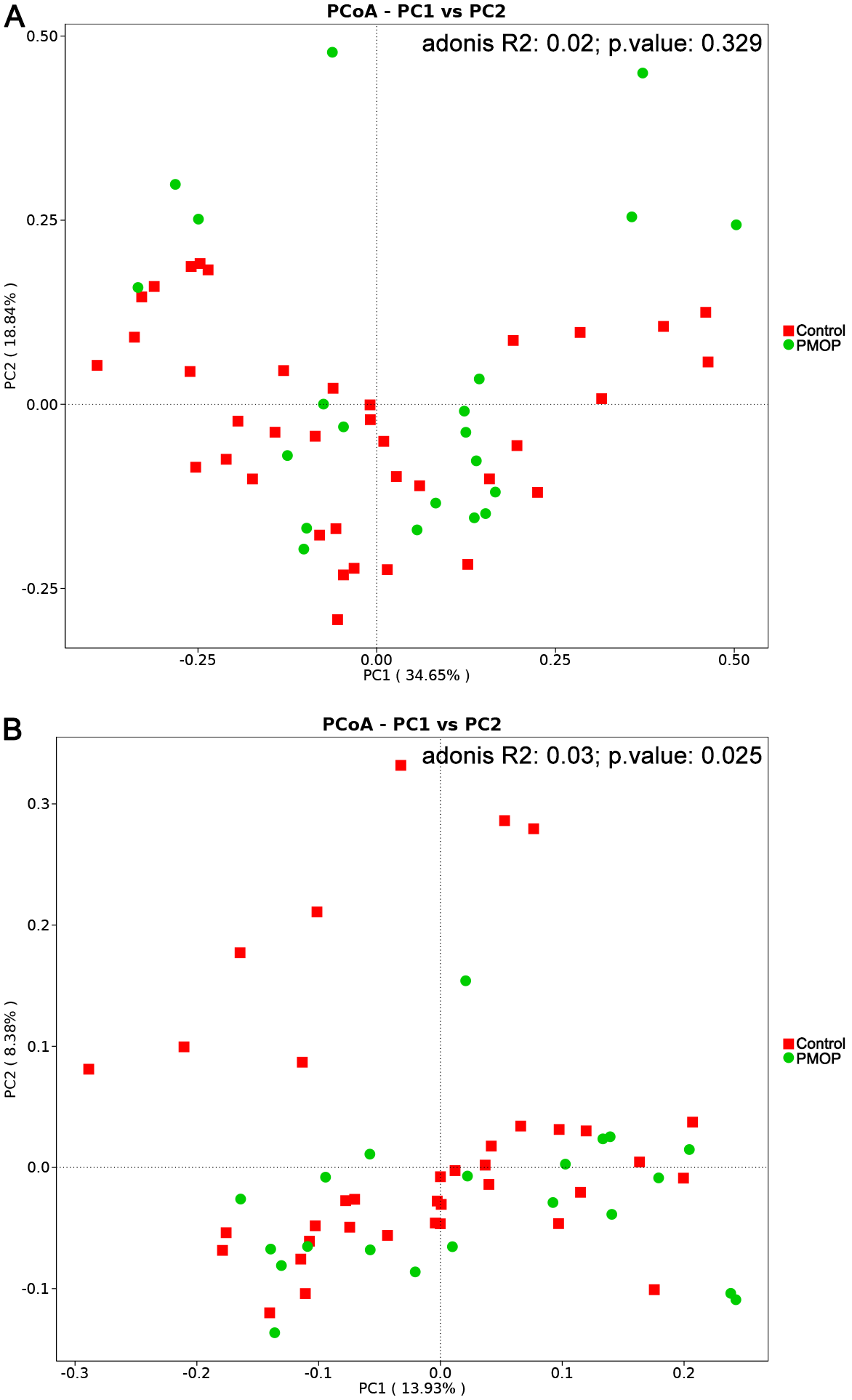

Supplement: Supplementary file 2 [file Image_2.TIF]

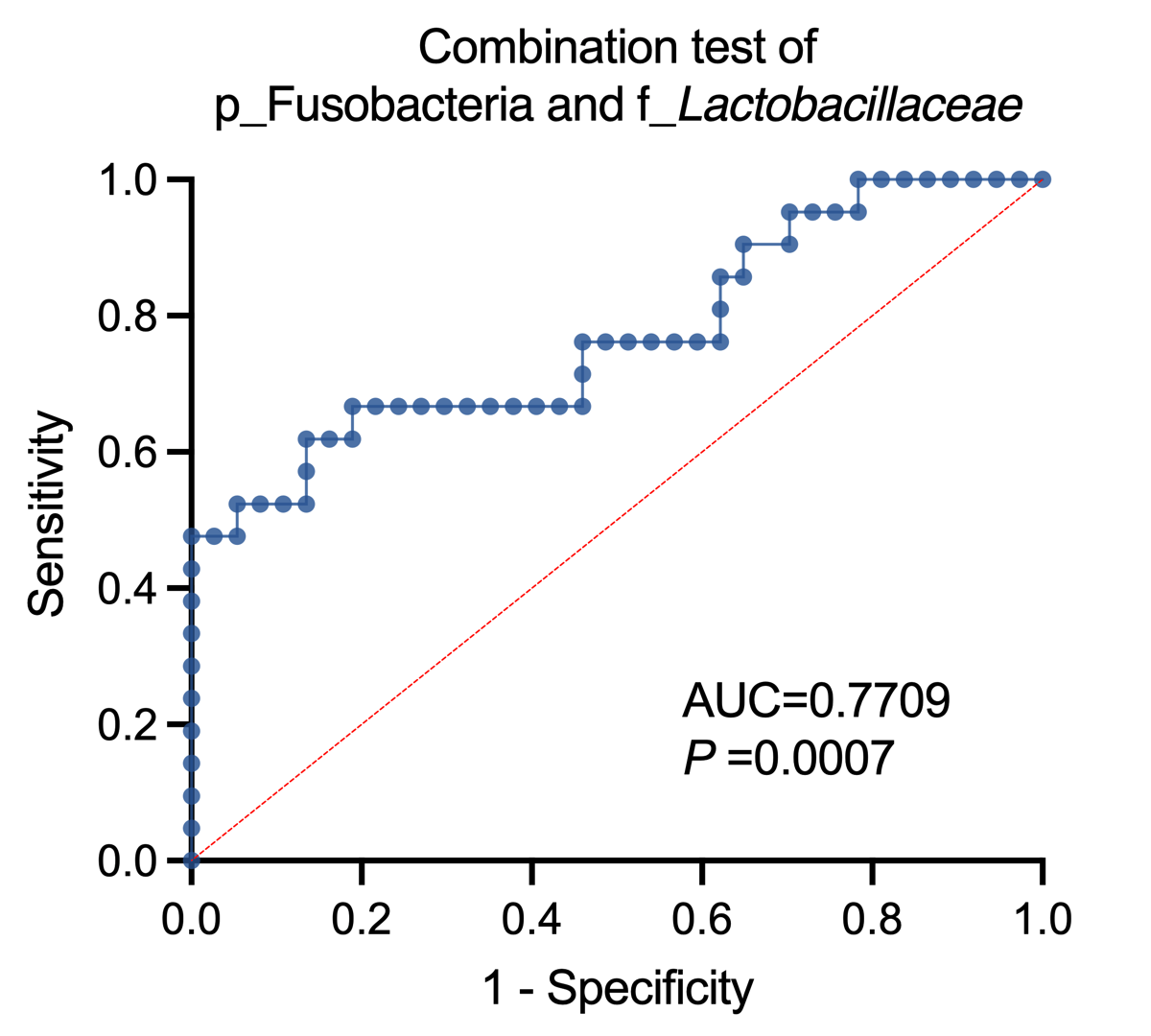

Supplement: Supplementary file 3 [file Image_3.TIFF]
