# Supplementary material for: Identifying microbial signatures for patients with postmenopausal osteoporosis using gut microbiota analyses and feature selection approaches
Source: Front Microbiol. 2023 Apr 3;14:1113174. doi: 10.3389/fmicb.2023.1113174 (PMC10106639; doi:10.3389/fmicb.2023.1113174)
Supplement: Supplementary file 4 [file Table_1.DOCX]

**Legends of Supplementary Tables and Figures**

**Table S1** Statistical results between PMOP and control groups in alpha diversity indices

| **Indices** | **Total (n=58)** | **PMOP (n=21)** | **Control (n=37)** | ***P* value** |
| --- | --- | --- | --- | --- |
| Observed_species | 299.76 ± 49.38 | 292.67 ± 50.47 | 303.78 ± 48.99 | 0.3276 |
| Shannon | 5.02 ± 0.58 | 5.04 ± 0.57 | 5.01 ± 0.60 | 0.8223 |
| Simpson | 0.93 ± 0.03 | 0.93 ± 0.03 | 0.92 ± 0.03 | 0.4406 |
| Chao1 | 359.63 ± 67.34 | 355.13 ± 72.87 | 362.19 ± 64.89 | 0.6188 |
| Ace | 364.01 ± 59.20 | 360.11 ± 60.10 | 366.22 ± 59.41 | 0.6648 |

The *P* value was obtained from Wilcoxon test in R.

**Table S2** Gut microbiota compositions and statistical results between the two groups

| **Taxonomy** | | **Total** | **PMOP** | **Control** | ***P* value** |
| --- | --- | --- | --- | --- | --- |
| **Phylum** | Firmicutes | 0.7116 | 0.6984 | 0.7190 | 0.8979 |
|  | Bacteroidetes | 0.1491 | 0.1386 | 0.1551 | 0.6302 |
|  | Actinobacteria | 0.1002 | 0.1187 | 0.0897 | 0.3766 |
|  | Proteobacteria | 0.0359 | 0.0387 | 0.0343 | 0.7340 |
|  | Verrucomicrobia | 0.0013 | 0.0033 | 0.0001 | 0.2992 |
|  | Tenericutes | 0.0008 | 0.0008 | 0.0008 | 0.6542 |
|  | Fusobacteria | 0.0005 | 0.0008 | 0.0003 | **0.0395** |
|  | unidentified_Bacteria | 0.0004 | 0.0004 | 0.0004 | 0.9547 |
|  | Melainabacteria | 0.0001 | 0.0000 | 0.0001 | 0.4447 |
|  | Oxyphotobacteria | 0.0000 | 0.0000 | 0.0000 | 0.8904 |
|  | Others | 0.0002 | 0.0002 | 0.0002 | Not report |
| **Class** | Clostridia | 0.6365 | 0.5869 | 0.6646 | 0.3346 |
|  | Bacteroidia | 0.1491 | 0.1386 | 0.1551 | 0.6302 |
|  | unidentified_Actinobacteria | 0.0975 | 0.1158 | 0.0872 | 0.3943 |
|  | Bacilli | 0.0455 | 0.0818 | 0.0249 | **0.0751** |
|  | Gammaproteobacteria | 0.0343 | 0.0372 | 0.0327 | 0.4311 |
|  | Negativicutes | 0.0211 | 0.0212 | 0.0211 | 0.1819 |
|  | Erysipelotrichia | 0.0085 | 0.0086 | 0.0084 | **0.0863** |
|  | Coriobacteriia | 0.0026 | 0.0029 | 0.0025 | 0.6331 |
|  | Deltaproteobacteria | 0.0013 | 0.0012 | 0.0014 | 0.3346 |
|  | Verrucomicrobiae | 0.0013 | 0.0033 | 0.0001 | 0.1623 |
|  | Others | 0.0022 | 0.0025 | 0.0020 | Not report |
| **Order** | Clostridiales | 0.6364 | 0.5869 | 0.6646 | 0.3266 |
|  | Bacteroidales | 0.1491 | 0.1385 | 0.1551 | 0.6302 |
|  | Bifidobacteriales | 0.0969 | 0.1150 | 0.0867 | 0.3943 |
|  | Lactobacillales | 0.0454 | 0.0816 | 0.0248 | **0.0700** |
|  | Enterobacteriales | 0.0292 | 0.0320 | 0.0276 | 0.5333 |
|  | Selenomonadales | 0.0211 | 0.0212 | 0.0211 | 0.1846 |
|  | Erysipelotrichales | 0.0085 | 0.0086 | 0.0084 | **0.0893** |
|  | unidentified_Gammaproteobacteria | 0.0041 | 0.0040 | 0.0041 | 0.2506 |
|  | Coriobacteriales | 0.0026 | 0.0029 | 0.0025 | 0.5989 |
|  | Verrucomicrobiales | 0.0013 | 0.0033 | 0.0001 | 0.6292 |
|  | Others | 0.0054 | 0.0060 | 0.0050 | Not report |
| **Family** | Ruminococcaceae | 0.3160 | 0.2564 | 0.3499 | **0.0332** |
|  | Lachnospiraceae | 0.2531 | 0.2399 | 0.2606 | 0.6648 |
|  | Bifidobacteriaceae | 0.0969 | 0.1150 | 0.0867 | 0.3943 |
|  | Bacteroidaceae | 0.0911 | 0.0796 | 0.0977 | 0.5175 |
|  | Prevotellaceae | 0.0378 | 0.0443 | 0.0342 | 0.8524 |
|  | unidentified_Clostridiales | 0.0294 | 0.0400 | 0.0234 | **0.0751** |
|  | Lactobacillaceae | 0.0293 | 0.0684 | 0.0071 | **0.0791** |
|  | Enterobacteriaceae | 0.0292 | 0.0320 | 0.0276 | 0.5175 |
|  | Veillonellaceae | 0.0184 | 0.0203 | 0.0174 | 0.5878 |
|  | Streptococcaceae | 0.0123 | 0.0102 | 0.0135 | 0.5602 |
|  | Others | 0.0863 | 0.0941 | 0.0819 | Not report |
| **Genus** | Faecalibacterium | 0.1945 | 0.1664 | 0.2104 | 0.2473 |
|  | Bifidobacterium | 0.0969 | 0.1150 | 0.0867 | 0.3943 |
|  | Bacteroides | 0.0912 | 0.0796 | 0.0977 | 0.5103 |
|  | Agathobacter | 0.0749 | 0.0687 | 0.0784 | 0.9488 |
|  | Blautia | 0.0642 | 0.0574 | 0.0681 | 0.5000 |
|  | unidentified_Lachnospiraceae | 0.0362 | 0.0354 | 0.0366 | 0.6447 |
|  | Lactobacillus | 0.0293 | 0.0684 | 0.0071 | **0.0834** |
|  | unidentified_Clostridiales | 0.0283 | 0.0397 | 0.0218 | **0.0415** |
|  | Streptococcus | 0.0117 | 0.0101 | 0.0126 | 0.4470 |
|  | Megamonas | 0.0094 | 0.0145 | 0.0066 | 0.5017 |
|  | Others | 0.3634 | 0.3448 | 0.3740 | Not report |
| **Species** | Ruminococcus_sp_5_1_39BFAA | 0.0436 | 0.0402 | 0.0456 | 0.4599 |
|  | Bifidobacterium_pseudocatenulatum | 0.0398 | 0.0558 | 0.0307 | 0.1705 |
|  | Bacteroides_vulgatus | 0.0339 | 0.0317 | 0.0352 | 0.6564 |
|  | Clostridium_disporicum | 0.0270 | 0.0379 | 0.0208 | **0.0493** |
|  | Bifidobacterium_adolescentis | 0.0202 | 0.0225 | 0.0188 | 0.3439 |
|  | Lactobacillus_mucosae | 0.0130 | 0.0283 | 0.0043 | 0.1001 |
|  | Bacteroides_caccae | 0.0075 | 0.0094 | 0.0064 | 0.1639 |
|  | Lactobacillus_salivarius | 0.0074 | 0.0197 | 0.0004 | **0.0087** |
|  | Bacteroides_plebeius | 0.0066 | 0.0049 | 0.0075 | 0.3369 |
|  | Bacteroides_eggerthii | 0.0040 | 0.0000 | 0.0062 | **0.0007** |
|  | Others | 0.7971 | 0.7495 | 0.8241 | Not report |

The *P* value was obtained from Wilcoxon test in R.

**Table S3** Spearman correlation analysis between gut microbial composition and BMD value/T-score

| **Taxonomy** | | **Lumbar spine** | | **Lumbar spine** | | **Total hip** | | **Total hip** | |
| --- | --- | --- | --- | --- | --- | --- | --- | --- | --- |
|  |  | **BMD value** | | **T score** | | **BMD value** | | **T score** | |
|  |  | ***ρ*** | ***P*** | ***ρ*** | ***P*** | ***ρ*** | ***P*** | ***ρ*** | ***P*** |
| **Phylun** | Firmicutes | 0.0797 | 0.5518 | 0.0777 | 0.5620 | 0.1944 | 0.1436 | 0.1892 | 0.1549 |
|  | Bacteroidetes | -0.1116 | 0.4044 | -0.1099 | 0.4115 | -0.1519 | 0.2551 | -0.1475 | 0.2693 |
|  | Actinobacteria | 0.0025 | 0.9854 | -0.0047 | 0.9718 | -0.1430 | 0.2844 | -0.1531 | 0.2511 |
|  | Proteobacteria | 0.1464 | 0.2727 | 0.1512 | 0.2571 | 0.0425 | 0.7513 | 0.0650 | 0.6277 |
|  | Verrucomicrobia | 0.2248 | 0.1898 | 0.2312 | 0.1808 | -0.0113 | 0.9332 | 0.0051 | 0.9698 |
|  | Tenericutes | -0.1641 | 0.2184 | -0.1579 | 0.2364 | -0.1026 | 0.4434 | -0.0771 | 0.5650 |
|  | Fusobacteria | -0.1692 | 0.2042 | -0.1669 | 0.2106 | -0.2454 | **0.0634** | -0.2680 | **0.0419** |
|  | unidentified_Bacteria | -0.0277 | 0.8365 | -0.0267 | 0.8420 | 0.0526 | 0.6947 | 0.0497 | 0.7110 |
|  | Melainabacteria | 0.1783 | 0.1806 | 0.1837 | 0.1675 | 0.1835 | 0.1680 | 0.1981 | 0.1360 |
|  | Oxyphotobacteria | 0.0331 | 0.8051 | 0.0433 | 0.7470 | -0.1218 | 0.3625 | -0.1150 | 0.3899 |
| **Class** | Clostridia | 0.1106 | 0.4087 | 0.1099 | 0.4114 | 0.3181 | **0.0150** | 0.3157 | **0.0158** |
|  | Bacteroidia | -0.1116 | 0.4044 | -0.1099 | 0.4115 | -0.1519 | 0.2551 | -0.1475 | 0.2693 |
|  | unidentified_Actinobacteria | 0.0026 | 0.9846 | -0.0045 | 0.9733 | -0.1572 | 0.2386 | -0.1675 | 0.2089 |
|  | Bacilli | -0.1372 | 0.3043 | -0.1377 | 0.3027 | -0.2046 | 0.1235 | -0.1800 | 0.1764 |
|  | Gammaproteobacteria | 0.2212 | 0.1951 | 0.2258 | 0.1884 | 0.0808 | 0.5463 | 0.1050 | 0.4328 |
|  | Negativicutes | 0.0854 | 0.5238 | 0.0817 | 0.5420 | -0.0406 | 0.7622 | -0.0538 | 0.6885 |
|  | Erysipelotrichia | -0.0245 | 0.8552 | -0.0188 | 0.8885 | -0.2618 | **0.0471** | -0.2770 | **0.0353** |
|  | Coriobacteriia | -0.0346 | 0.7965 | -0.0315 | 0.8146 | -0.0567 | 0.6726 | -0.0693 | 0.6052 |
|  | Deltaproteobacteria | -0.0492 | 0.7139 | -0.0387 | 0.7729 | 0.0203 | 0.8796 | 0.0041 | 0.9759 |
|  | Verrucomicrobiae | 0.2726 | 0.1384 | 0.2783 | 0.1344 | 0.0434 | 0.7461 | 0.0565 | 0.6733 |
| **Order** | Clostridiales | 0.1125 | 0.4006 | 0.1119 | 0.4030 | 0.3200 | **0.0143** | 0.3177 | **0.0151** |
|  | Bacteroidales | -0.1116 | 0.4044 | -0.1099 | 0.4115 | -0.1519 | 0.2551 | -0.1475 | 0.2693 |
|  | Bifidobacteriales | 0.0035 | 0.9792 | -0.0036 | 0.9788 | -0.1564 | 0.2410 | -0.1666 | 0.2112 |
|  | Lactobacillales | -0.1420 | 0.2875 | -0.1424 | 0.2862 | -0.2104 | 0.1129 | -0.1860 | 0.1621 |
|  | Enterobacteriales | 0.1149 | 0.3905 | 0.1184 | 0.3761 | -0.0337 | 0.8017 | -0.0084 | 0.9500 |
|  | Selenomonadales | 0.0870 | 0.5162 | 0.0832 | 0.5346 | -0.0423 | 0.7527 | -0.0555 | 0.6792 |
|  | Erysipelotrichales | -0.0240 | 0.8578 | -0.0182 | 0.8920 | -0.2665 | **0.0431** | -0.2824 | **0.0317** |
|  | unidentified_Gammaproteobacteria | 0.1672 | 0.2097 | 0.1715 | 0.1981 | 0.1108 | 0.4075 | 0.1239 | 0.3542 |
|  | Coriobacteriales | -0.0331 | 0.8051 | -0.0305 | 0.8204 | -0.0489 | 0.7153 | -0.0603 | 0.6527 |
|  | Verrucomicrobiales | 0.1500 | 0.2609 | 0.1548 | 0.2460 | -0.0612 | 0.6480 | -0.0388 | 0.7723 |
| **Family** | Ruminococcaceae | 0.0810 | 0.5455 | 0.0790 | 0.5555 | 0.1763 | 0.1855 | 0.2052 | 0.1222 |
|  | Lachnospiraceae | 0.1012 | 0.4499 | 0.1047 | 0.4339 | 0.3604 | **0.0055** | 0.3327 | **0.0107** |
|  | Bifidobacteriaceae | 0.0023 | 0.9865 | -0.0049 | 0.9709 | -0.1561 | 0.2421 | -0.1663 | 0.2123 |
|  | Bacteroidaceae | -0.1203 | 0.3683 | -0.1192 | 0.3730 | -0.1808 | 0.1744 | -0.1762 | 0.1858 |
|  | Prevotellaceae | -0.0916 | 0.4941 | -0.0883 | 0.5096 | -0.1072 | 0.4231 | -0.1140 | 0.3942 |
|  | unidentified_Clostridiales | -0.1645 | 0.2171 | -0.1619 | 0.2247 | -0.0602 | 0.6535 | -0.0605 | 0.6516 |
|  | Lactobacillaceae | -0.1379 | 0.3020 | -0.1383 | 0.3007 | -0.1937 | 0.1451 | -0.1481 | 0.2673 |
|  | Enterobacteriaceae | 0.1149 | 0.3903 | 0.1182 | 0.3770 | -0.0437 | 0.7447 | -0.0171 | 0.8989 |
|  | Veillonellaceae | 0.0265 | 0.8433 | 0.0223 | 0.8678 | -0.0904 | 0.5000 | -0.0948 | 0.4789 |
|  | Streptococcaceae | 0.0381 | 0.7762 | 0.0403 | 0.7640 | 0.0220 | 0.8698 | 0.0219 | 0.8706 |
| **Genus** | Faecalibacterium | -0.0365 | 0.7855 | -0.0381 | 0.7766 | 0.0552 | 0.6805 | 0.0718 | 0.5924 |
|  | Bifidobacterium | 0.0026 | 0.9846 | -0.0045 | 0.9733 | -0.1572 | 0.2386 | -0.1675 | 0.2089 |
|  | Bacteroides | -0.1202 | 0.3688 | -0.1190 | 0.3736 | -0.1785 | 0.1802 | -0.1738 | 0.1919 |
|  | Agathobacter | 0.0203 | 0.8796 | 0.0172 | 0.8982 | 0.1560 | 0.2424 | 0.1623 | 0.2235 |
|  | Blautia | 0.0916 | 0.4943 | 0.0941 | 0.4824 | 0.2441 | **0.0648** | 0.2165 | 0.1026 |
|  | unidentified_Lachnospiraceae | -0.0829 | 0.5360 | -0.0760 | 0.5708 | 0.1953 | 0.1419 | 0.1886 | 0.1562 |
|  | Lactobacillus | -0.1367 | 0.3063 | -0.1367 | 0.3062 | -0.1949 | 0.1427 | -0.1479 | 0.2678 |
|  | unidentified_Clostridiales | -0.1851 | 0.1642 | -0.1814 | 0.1730 | -0.0745 | 0.5783 | -0.0803 | 0.5488 |
|  | Streptococcus | 0.0222 | 0.8684 | 0.0227 | 0.8656 | 0.0165 | 0.9019 | 0.0164 | 0.9029 |
|  | Megamonas | -0.1714 | 0.1983 | -0.1781 | 0.1809 | -0.2283 | **0.0847** | -0.2409 | **0.0685** |
| **Species** | Ruminococcus_sp_5_1_39BFAA | 0.0988 | 0.4608 | 0.1015 | 0.4484 | 0.2360 | **0.0745** | 0.2054 | 0.1219 |
|  | Bifidobacterium_pseudocatenulatum | -0.0939 | 0.4834 | -0.1006 | 0.4525 | -0.2769 | **0.0353** | -0.2887 | **0.0279** |
|  | Bacteroides_vulgatus | -0.1231 | 0.3571 | -0.1227 | 0.3590 | -0.1586 | 0.2345 | -0.1467 | 0.2717 |
|  | Clostridium_disporicum | -0.2162 | 0.1031 | -0.2129 | 0.1086 | -0.0733 | 0.5846 | -0.0789 | 0.5560 |
|  | Bifidobacterium_adolescentis | -0.0256 | 0.8488 | -0.0290 | 0.8291 | -0.2121 | 0.1099 | -0.2160 | 0.1034 |
|  | Lactobacillus_mucosae | -0.0472 | 0.7248 | -0.0525 | 0.6955 | -0.1616 | 0.2257 | -0.1073 | 0.4225 |
|  | Bacteroides_caccae | -0.0125 | 0.9257 | -0.0134 | 0.9207 | -0.0249 | 0.8529 | -0.0193 | 0.8858 |
|  | Lactobacillus_salivarius | -0.3476 | **0.0075** | -0.3466 | **0.0077** | -0.4258 | **0.0009** | -0.4118 | **0.0013** |
|  | Bacteroides_plebeius | 0.0087 | 0.9482 | 0.0085 | 0.9492 | -0.0110 | 0.9344 | -0.0239 | 0.8586 |
|  | Bacteroides_eggerthii | 0.3192 | **0.0146** | 0.3104 | **0.0177** | 0.3114 | **0.0173** | 0.2946 | **0.0248** |

**Figure S1** The species accumulation box-plot of the 58 samples.

**Figure S2** Sample clustering between PMOP and control groups. (**A**) PCoA plot based on the weighted UniFrac distance of samples from PMOP vs. control group. (**B**) PCoA plot based on the unweighted UniFrac distance of samples from PMOP vs. control group. The PerMANOVA tests were performed using “adonis” function in R.

**Figure S3** ROC curves of combination tests of p_Fusobacteria & f_*Lactobacillaceae* as microbial biomarkers in distinguishing PMOP from control group.
